# Molecular profiling of patient-derived breast cancer xenografts

**DOI:** 10.1186/bcr3095

**Published:** 2012-01-16

**Authors:** Fabien Reyal, Charlotte Guyader, Charles Decraene, Carlo Lucchesi, Nathalie Auger, Franck Assayag, Ludmilla De Plater, David Gentien, Marie-France Poupon, Paul Cottu, Patricia De Cremoux, Pierre Gestraud, Anne Vincent-Salomon, Jean-Jacques Fontaine, Sergio Roman-Roman, Olivier Delattre, Didier Decaudin, Elisabetta Marangoni

**Affiliations:** 1 Department of Surgery and UMR144 Institut Curie, Molecular Oncology Team, Institut Curie, 26 rue d'Ulm, 75005 Paris, France; 2 Translational Research Department, Institut Curie, 26 rue d'Ulm, 75005 Paris, France; 3 CNRS, UMR144, Institut Curie, 26 rue d'Ulm, 75005 Paris, France; 4 INSERM U830, Institut Curie, 26 rue d'Ulm, 75005 Paris, France; 5 Affymetrix platform, Translational Research Department, Institut Curie, 26, rue d'Ulm, 75005 Paris, France; 6 Department of Medical Oncology, Institut Curie, 26 rue d'Ulm, 75005 Paris, France; 7 Department of Tumor Biology, Institut Curie, 26 rue d'Ulm, 75005 Paris, France; 8 Bioinformatics, Institut Curie, 26 rue d'Ulm, 75005 Paris, France; 9 National Veterinary School of Alfort, 7, Av. du Général de Gaulle, 94704 Maisons Alfort Cedex, France

**Keywords:** Breast cancer, xenograft, genomic and expression profiles

## Abstract

**Introduction:**

Identification of new therapeutic agents for breast cancer (BC) requires preclinical models that reproduce the molecular characteristics of their respective clinical tumors. In this work, we analyzed the genomic and gene expression profiles of human BC xenografts and the corresponding patient tumors.

**Methods:**

Eighteen BC xenografts were obtained by grafting tumor fragments from patients into Swiss nude mice. Molecular characterization of patient tumors and xenografts was performed by DNA copy number analysis and gene expression analysis using Affymetrix Microarrays.

**Results:**

Comparison analysis showed that 14/18 pairs of tumors shared more than 56% of copy number alterations (CNA). Unsupervised hierarchical clustering analysis showed that 16/18 pairs segregated together, confirming the similarity between tumor pairs. Analysis of recurrent CNA changes between patient tumors and xenografts showed losses in 176 chromosomal regions and gains in 202 chromosomal regions. Gene expression profile analysis showed that less than 5% of genes had recurrent variations between patient tumors and their respective xenografts; these genes largely corresponded to human stromal compartment genes. Finally, analysis of different passages of the same tumor showed that sequential mouse-to-mouse tumor grafts did not affect genomic rearrangements or gene expression profiles, suggesting genetic stability of these models over time.

**Conclusions:**

This panel of human BC xenografts maintains the overall genomic and gene expression profile of the corresponding patient tumors and remains stable throughout sequential *in vivo *generations. The observed genomic profile and gene expression differences appear to be due to the loss of human stromal genes. These xenografts, therefore, represent a validated model for preclinical investigation of new therapeutic agents.

## Introduction

Breast cancer (BC) is the most commonly diagnosed cancer and remains the leading cause of worldwide cancer-related death in women [1]. A better understanding of BC biology is essential in order to identify new targeted therapies and tumors with molecular profiles that will respond to the targeted treatment. Gene expression profiling of invasive BC has defined three main tumor subtypes with very specific features (Luminal, Basal, human epidermal growth factor receptor 2 (*HER2)*) [2]. It is now common knowledge that the pathologic characteristics, array comparative genomic hybridization (aCGH) profiles, gene and miRNA expression profiles and activated pathways are radically different among these subtypes, supporting the view that BC is a disease composed of very different and independent molecular subgroups. These subtypes have also been shown to differ in terms of clinical presentation (that is, differences in axillary lymph node involvement, local and regional recurrence, metastatic patterns and overall prognosis), and in their sensitivity to systemic treatment [3].

Preclinical experimental models that reproduce the heterogeneity of this disease have become a major challenge in order to investigate the biology of each BC subtype and evaluate new targeted therapies. Such models are necessary to examine the treatment efficacy of potential new therapies and some have already contributed to the development of new human therapeutics [4,5]. However, most of the existing *in vivo *models used for preclinical trials of anticancer drugs are based on a limited number of cell lines previously isolated from human tumors and selected by cell culture prior to implantation in immunodeficient animals. These models do not reproduce the architecture of the primary tumors [6,7]. In contrast, tumor xenografts obtained by engraftment of tumor samples transplanted directly into animals seems to be able to reduce the biologic distance between the original patient tumor and the *in vivo *model. We have previously published a paper describing a large panel of BC xenografts that maintain the cell differentiation, morphology, architecture, vasculature, peripheral growth and some of the molecular features of the original patient's tumor [8].

However, new aberrations are expected to appear in xenografted tumors because of the selection pressure operated by the host animal, the loss of human stroma and the intrinsic genetic instability of breast tumors.

To address these issues, we compared the genomic (that is, aCGH) profiles and gene expression profiles of BC xenografts with their corresponding primary tumors. We then evaluated tumor stability in human BC xenografts transplanted serially over several years, by comparing their profiles at early and late *in vivo *generations.

Genomic analyses showed that BC xenografts reflect the genomic profile of the patient's tumors, with additional DNA gains and losses. Gene expression profile analysis showed dynamic variations between tumor pairs (xenograft and primary tumor), with recurrent changes in the expression of a small group of stroma-related genes.

These data suggest that BC xenografts maintain the overall genetic profile of the original tumors, with additional changes that could be explained by adaptation of tumor cells to the new host.

## Materials and methods

### Establishment of tumor xenografts

Tumor specimens were obtained from BC patients with their informed consent. Tumor fragments were removed during surgery, as previously described [8]. Briefly, fresh tumor fragments were grafted subcutaneously into the interscapular fat pad of female Swiss nude mice under anesthesia. Mice were kept in pathogen-free animal housing (Institut Curie) and received estrogen (8 μg/mL) diluted in drinking water. Xenografts appeared at the graft site two to eight months after grafting. They were subsequently transplanted from mouse to mouse. The experimental protocol and animal housing were in accordance with institutional guidelines as proposed by the French Ethics Committee (Agreement B75-05-18, France).

### Histology and immunohistochemistry

The morphology of patient tumor tissues was compared with that of the corresponding xenografts by examining paraffin-embedded sections according to standard protocols [8]. Tumors were removed from mice and immediately fixed in 10% formaldehyde solution for immunohistologic examination.

Immunostaining was performed according to previously published protocols [9]. Briefly, 4 μm tissue sections were prepared from a representative sample of the tumor. After rehydration and antigen retrieval in citrate buffer (10 mM, pH 6.1), tissue sections were stained for estrogen receptor (ER), progesterone receptor (PR) and ERBB2/neu (HER2). Staining was revealed with the Vectastain Elite ABC peroxidase mouse Immunoglobulin G kit (Vector, Burlingame, CA, USA) using diaminobenzidine (Dako A/S, Glostrup, Denmark) as chromogen. Positive nuclear staining for ER and PR was recorded according to standardized guidelines. ER (clone 6F11; 1/200; Novocastra, Rungis, France), PR (PR; clone 1A6; 1/200; Novocastra) and ERBB2 (clone CB11; 1/1,000; Novocastra) expression was evaluated. For ERBB2, only membranous staining was evaluated, as previously defined [10].

### Array comparative genomic hybridization

Genomic DNA was extracted as previously published [11]. Co-hybridization was performed between extracted DNA (primary BC or corresponding xenograft) and normal DNA. Genome-wide resources of 3,261 or 5,244 fluorescence *in situ *hybridization-mapped sequenced bacterial artificial chromosome (BAC) and P1-derived artificial chromosome (PAC) clones were represented as immobilized DNA targets on glass slides, allowing a mean resolution of 0.5 Mb throughout the genome. Each clone was spotted in quadruplicate on slides prepared by Integragen™ (Evry, France). DNA samples, each 1.5 μg, were digested with DpnII enzyme (Ozyme, Saint-Quentin-en-Yvelines, France) and labeled with random priming using a Bioprime DNA labeling kit (Life Technologies, Villebon sur Yvette, France) with the appropriate cyanine dye (Cy3 or Cy5; Perkin-Elmer, Courtaboeuf, France). The control and test DNAs were co-precipitated with Cot-1 DNA (Life Technologies, Villebon sur Yvette, France), denatured and re-suspended in hybridization buffer. After 24 hours of hybridization, slides were washed with sodium dodecyl sulfate buffer and saline citrate, dried and scanned with a GenePix 4000B scanner (Axon Instruments Inc., Union City, CA, USA). Image analysis was performed with GenePix 5.1 software (Axon) [12]. Any BAC with less than two replicates flagged for not meeting qualitative spot criteria was excluded. Normalization was performed with the MANOR algorithm [12]. Spots showing a low signal-to-noise ratio or poor replicate consistency were discarded. Status assignment (loss, normal, gain and amplification of chromosome copy number) was performed by using the GLAD algorithm [13].

Hierarchical clustering was performed on profiles based on probe status. The group average was used as the similarity measure and the Pearson algorithm was used as the agglomerative method. Separation into groups was proposed on the basis of the structure of the dendrogram. Data visualization and computation of clustering were performed according to the VAMP (Vizualization and Analysis of Molecular Profiles) analysis procedure [12]. Pearson's correlation coefficient was calculated on altered clones based on their clone status (clone status, that is, GNL: gain, normal, loss). For analysis of GNL differences, chromosomal segments were defined as genomic regions with a constant copy number for all samples.

### RNA extraction

Prior to RNA extraction, a tissue section from the tumor fragments was stained with hematoxylin and eosin to evaluate tumor cellularity. All tumors analyzed comprised more than 40% of tumor cells on the tissue section. Total RNA was isolated from 15-65 mg of frozen tissue using TRIzol reagent (Invitrogen, Cergy-Pontoise, France) according to the manufacturer's instructions. The RNA concentration was measured by absorbance at 260 nm. The quality of each RNA sample was determined on the Agilent 2100 bioanalyzer (Agilent Technologies, Palo Alto, CA, USA. RNA was processed on chips only when the following criteria were met: RIN (a measurement of RNA quality) ≥ 7, (28S/18S) ≥ 1.4, (260 nm/230 nm) ≥ 1.8, and (260 nm/280 nm) ≥ 1.8.

### Gene expression analysis

#### Affymetrix HGU133 Plus 2.0

The concentration and integrity/purity of each RNA sample were measured using RNA 6000 LabChip kit (Agilent) and the Agilent 2100 bioanalyzer. The DNA microarrays used in this study were the Human Genome U133 Plus 2.0 array containing 54,675 probe sets (Affymetrix, Santa Clara, CA, USA). One hundred nanograms of total RNA were amplified and labeled according to the Affymetrix 3'IVT express protocol. Each batch of targets included an MAQC A sample to control for target preparation and hybridization. Targets were validated according to yield and size of RNA, usually obtained at the Institut Curie molecular biology facility. Targets were hybridized on human and mouse microarrays. Chips were washed and stained on a fluidic station according to the manufacturer's protocol and were scanned using an Affymetrix GCS3000 scanner. Microarray quality control assessment was performed using the R AffyPLM and SimpleAffy packages available from the Bioconductor web site. Relative Log Expression, Normalized Unscaled Standard Errors, scaling factor, percentage of "present" calls, 3'/5' ratio and average background tests were applied to determine the quality of each experiment. Chip pseudo-images were produced to assess artifacts on arrays that failed to pass the previous quality control tests. Selected arrays were normalized according to the GC-RMA normalization procedure [14]. Raw data can be obtained from the Institut Curie Microarray Database [15].

### Statistical analysis

#### Quality control analysis

Thirty-two xenograft and primary tumor samples and two universal RNA were hybridized. Twenty-eight of the 32 gene expression microarrays were deemed to be of sufficient quality. Considering that an expression signal below a cut-off of 3.5 after GC-RMA normalization cannot be distinguished from noise or missing signal, the present analysis was based exclusively on probe sets with a signal level less than 3.5 in no more than 85% of the samples analyzed: 29,683 out of 54,675 probe sets were included in the analysis.

### Molecular subtype classification

Hu *et al*. defined and validated the centroids of 306 genes to discriminate between five previously identified BC molecular subtypes (Luminal A and B, Basal, HER2 positive, Normal) [16]. The UniGene ID (Build204) gene list was matched to the HG-U133 Plus 2.0 Affymetrix^© ^platform annotation. Each sample was assigned to the nearest subtype/centroid as determined by the highest Spearman rank order correlation between the gene expression values of the molecular subtype probe sets and the five subtype centroids. A sample with a maximum correlation score less than 0.2 was considered to be unclassified.

### Differential expression analysis

The gene expression profiles of the pairs (xenograft and corresponding primary tumor; xenograft at different tumor passages) were assumed to be similar (null hypothesis). The scatter plots of the whole gene expression data set for pairs of xenografts and corresponding primary tumors and pairs of xenografts from different passages confirmed this hypothesis. A linear regression model was fitted to analyze the variation in gene expression of each pair (xenograft and corresponding primary tumor or xenograft after several generations). Linear regression models were built to define the 5th and 95th percentiles of the residual distribution. Normality testing was limited to observation of the density plot and a quantile-quantile plot of the residual values. A residual above the 95th percentile or below the 5th percentile was considered to be an outlier.

### Gene ontology analysis

This analysis was performed to determine whether specific gene sets (that is, functional groups) were overrepresented in the various gene lists. The DAVID (Database for Annotation, Visualization and Integrated Discovery) Gene Ontology web site was used to test the significance of enrichment in specific gene ontology annotations [17].

## Results

### Histologic and immunohistochemical analysis of tumors

**Figure 1:**
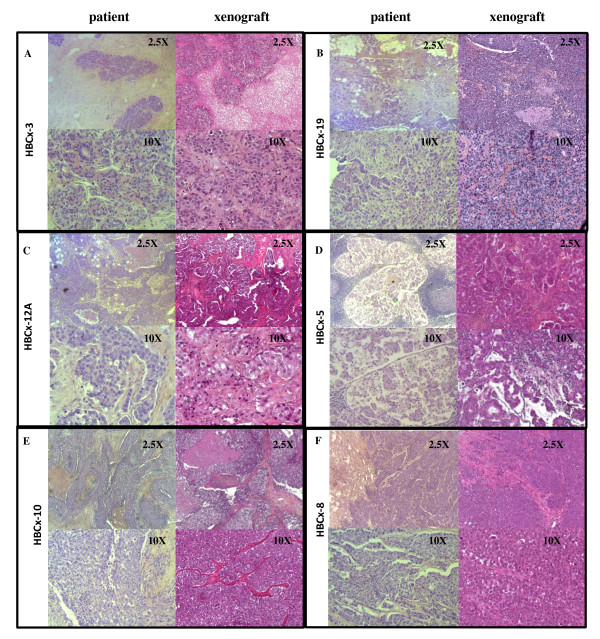
**Histology and IHC analysis of primary tumors and xenografts**. Representative hematoxylin-eosin or hematoxylin-eosin-saffron stained sections of patient tumors and xenografts. **A**, luminal tumor HBC(x)-3. **B**, lobular tumor HBCx-19. **C**, triple-negative tumor HBC(x)-12A. **D**, HER2+ tumor HBCx-5. **E**, triple-negative tumor HBC(x)-10. **F**, triple-negative tumor HBC(x)-8. HBC, human breast cancer, HBC-n, HBC primary tumor n, HBCx-n, HBC xenograft tumor n, HER2+, human epidermal growth factor receptor 2 positive, IHC, immunohistochemistry.

A preliminary histologic analysis of xenografts compared their morphology and pathological classification (based on HER2, ER and PR receptor expression) with those of the primary tumors. Results based on immunohistochemistry data are summarized in Table 1 and have been previously published in part [8,18]. Figure 1 illustrates the histology of patient- and xenograft-derived tumors for each of the following BC subtypes: luminal human breast cancer xenograft (HBCx-3), triple-negative (HBCx-8, HBCx-12A and HBCx-10), HER2+ (HBCx-5), and lobular (HBCx-19). The primary HBC-3 BC was an infiltrating ductal adenocarcinoma organized in cell cords with a small *in situ *component. The xenografted tumors showed a similar architecture with an abundant stromal component. The triple-negative HBCx-8 and HBCx-10 tumors were diagnosed as infiltrating ductal carcinoma with a trabecular architecture that was reproduced in the xenograft. The HBCx-12A was a poorly differentiated ductal carcinoma with a large number of mitotic figures and large cells with abundant cytoplasm. Images at a magnification of 2.5 × of the patient's luminal HBCx-3 and HER2+ HBCx-5 tumors showed a high content of stromal component (Figure 1A, D). Organization of stroma around the tumor cell nests in HER2+ tumors was reproduced in the xenograft.

**Table 1:** Histopathologic features of breast cancer xenografts and genomic correlation between primary tumor and xenograft

Tumor xenograft	Phenotype (IHC) Patient/xenograft	Histology	Pearson's correlation coefficient
**HBCx-3**	ER+/ER+	IDC	0.76
**HBCx-5**	HER2+/HER2+	IDC	0.35
**HBCx-6**	TN/TN	IDC	0.60
**HBCx-7**	TN/TN	ILC	0.23
**HBCx-8**	TN/TN	IDC	0.82
**HBCx-10**	TN/TN	IDC	0.57
**HBCx-11**	TN/TN	IDC	0.65
**HBCx-12A**	TN/TN	IDC	0.66
**HBCx-13A**	HER2+/HER2+	IDC	0.86
**HBCx-15**	TN/TN	IDC	0.57
**HBCx-16**	TN/TN	IDC	0.46
**HBCx-17**	TN/TN	IDC	0.39
**HBCx-20**	ER+/ER+	IDC	0.87
**HBCx-21**	ER+PR+/ER+PR+	IDC	0.86
**HBCx-22**	ER+ PR+/ER+PR+	IDC	0.68
**HBCx-23**	TN/TN	IDC	0.80
**HBCx-24**	TN/TN	IDC	0.77
**HBCx-31**	TN/TN	IDC	0.86

ER, estrogen receptor; HBC, human breast cancer; HBC-n, HBC primary tumor n; HBCx-n, HBC xenograft tumor n; HER, human epidermal growth factor receptor; IDC, invasive ductal carcinoma; IHC, immunohistochemistry; ILC; invasive lobular carcinoma; PR, progesterone receptor; TN, triple-negative. List of xenografts used in the study according to the breast cancer subtype. Expression of HER2; ER and PR was determined by IHC. Pearson's correlation coefficients between pairs of patient tumors and xenografts were calculated between the clone status (Gain, Normal, Loss) of the same probe.

### Array CGH analysis of xenografts and corresponding patient tumors

To evaluate the similarity of genomic profiles between the tumors and their corresponding xenografts, a BAC aCGH analysis was performed on 18 pairs, of which 2 were HER2+, 4 were ER positive and 12 were triple-negative (Table 1).

Copy number alterations (CNA) of each pair of patient tumors and xenografts were compared by calculating Pearson's correlation coefficients R. Fourteen pairs had a correlation coefficient greater than 0.50, indicating similarity (Table 1). The correlation coefficient was less than 0.5 in four pairs: one pair was HER2+ and three were triple-negative. No correlation with the percentage of high quality clones analyzed was identified (data not shown).

**Figure 2:**
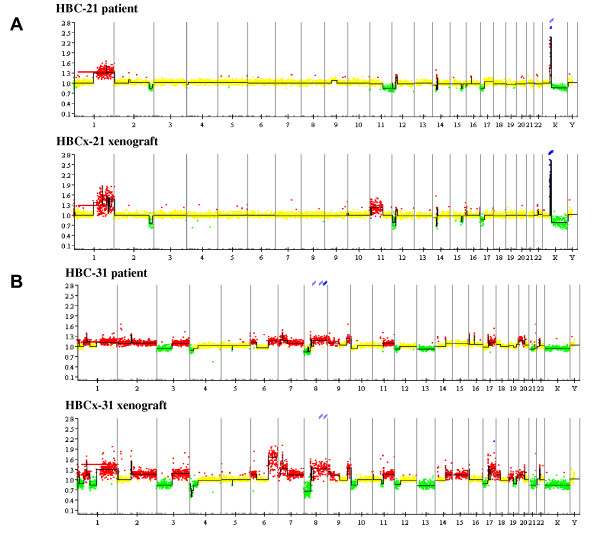
**Array CGH profiles of patient tumors (top) and xenografts (bottom) compared with normal DNA**. Recurrence of copy number alterations is plotted on the y-axis and each probe is aligned along the x-axis in chromosomal order. Loss, gain or amplification of gene copy numbers are depicted in green, red and blue, respectively. **A**, array CGH profile of a luminal tumor. **B**, array CGH profile of a triple-negative tumor. CGH, comparative genomic hybridization, HBC, human breast cancer, HBC-n, HBC primary tumor n, HBCx-n, HBC xenograft tumor n.

Two examples of paired aCGH profiles are shown in Figure 2. In Figure 2A, both the primary ER positive tumor and the xenograft displayed a slightly altered profile. Most of the alterations were conserved in the xenografts, such as the 1q gain, Xp22 amplification, 2q loss, and alterations in 14p, 15q, and 17. A gain in the 11p region was observed in the xenograft but not in the original tumor. Profiles of the triple-negative tumors are shown in Figure 2B. Most of the chromosomes were affected by genomic alterations. Large genomic gains were observed in almost all chromosomes. Three large genomic losses, in chromosomes 3, 13 and X, were conserved in the xenograft. Slight amplifications of genomic alterations were observed in the xenograft profile, for example, in chromosomes 1, 6, 7 and 17. Non-altered regions were fairly similar in both profiles.

### Clustering of aCGH data set

**Figure 3:**
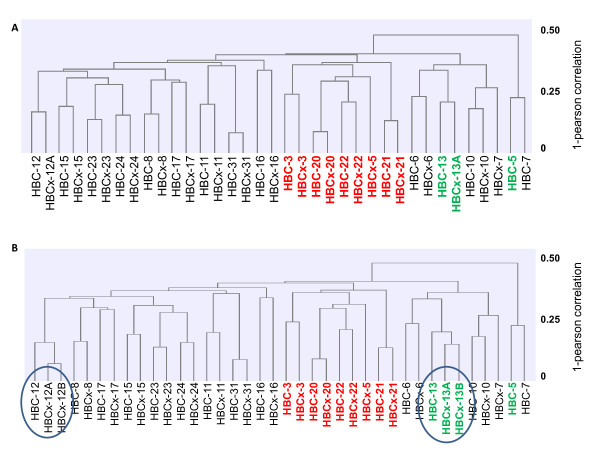
**Clustering of array CGH data set**. The length of each horizontal dendrogram arm indicates the degree of correlation between the various specimens, ranging from 0% to 100% correlation. The shorter the dendrogram arm, the greater the degree of correlation. **A**, unsupervised hierarchical clustering analysis of copy number alterations on the whole sample set (18 pairs of primary tumors and corresponding xenografts). **B**, hierarchical clustering analysis, including the metastasis-derived xenografts HBCx-13B and HBCx-12B (indicated with circles). CGH, comparative genomic hybridization, HBC, human breast cancer, HBC-n, HBC primary tumor n, HBCx-n, HBC xenograft tumor n, HER2+, human epidermal growth factor receptor 2 positive. Red, luminal tumors; black, triple-negative tumors; green, HER2+ tumors.

**Figure 4:**
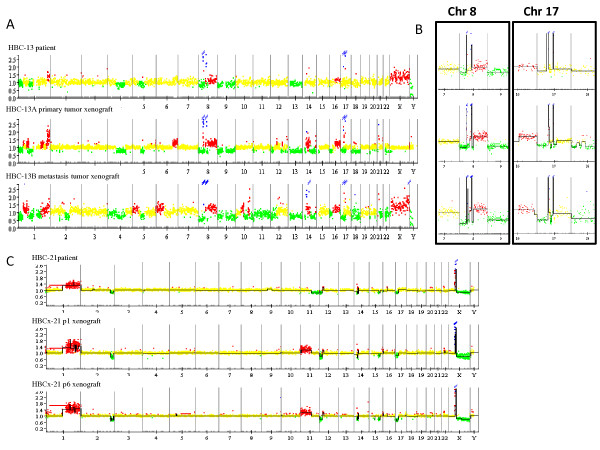
**Stability of CGH profiles between primary and metastasis profiles and throughout successive xenograft generations**. **A**, aCGH profile of the HBCx-13 tumors derived from a patient's primary breast tumor (upper), breast tumor-derived xenograft (middle), and axillary metastasis-derived xenograft (bottom). **B**, details of the aCGH profile of chromosome 17 amplicon (containing the HER2 oncogene amplification and chromosome 8 amplicon. **C**, aCGH profiles of the luminal HBC-21 patient tumor and xenografts from different *in vivo *tumor passages (p6 corresponding to a time lapse of 30 months after the first tumor engraftment). aCGH, array comparative genomic hybridization; HBC, human breast cancer; HBC-n, HBC primary tumor n; HBCx-n, HBC xenograft tumor n; HER2, human epidermal growth factor receptor 2.

Unsupervised hierarchical clustering analysis of CNA was performed on the whole sample set (18 pairs of primary tumors and corresponding xenografts) in order to determine whether tumors and xenografts were clonally related (Figure 3A). Sixteen of the 18 patient-xenograft pairs clustered together, indicating greater differences in CNA between primary tumors than between a primary tumor and its corresponding xenograft. Luminal and HER2+ BC subtypes clustered into two separate subgroups that were distinct from the triple-negative subgroup. In two cases, xenografts derived from the primary tumor and axillary lymph node from the same patient (HBCx-12A/B and HBCx-13A/B) clustered closely with the corresponding primary tumors (Figure 3B). This result highlights the fact that no major differences in CNA were observed between xenografts derived from breast tumors or the corresponding axillary node metastases. In addition, the two HBCx-12A/12B and HBCx-13A/13B pairs clustered into two small subclusters, indicating a higher degree of similarity between xenografts derived from primary tumors and metastases than between patient tumors and xenografts. An example of the similarity between primary tumor-derived xenografts and metastasis-derived xenografts is shown in Figure 4A. The Pearson correlation coefficients between the patient tumor and the corresponding xenograft were 0.86 and 0.82 (for a primary tumor-derived xenograft and a metastasis-derived xenograft, respectively). A very strong correlation was also observed between the two xenografts, with a correlation coefficient of 0.85. Importantly, the HER2 amplicon, localized on chromosome 17q21-22, was preserved in both the primary tumor-derived xenograft and the metastasis-derived xenograft (Figure 4B). A second amplicon, frequently associated with HER2 amplicons, localized on chromosome 8 (8p12-p11), was also found in the three genetic profiles.

To study the stability of xenografts in serial transplantation, the genetic profile of four xenografts was analyzed from different passages and compared to the profile of the original patient tumor. The genomic profiles of xenografts remained very stable throughout sequential *in vivo *passages. An example of aCGH profiles at different passages is shown in Figure 4C; the HBCx-21 profiles at p1 and p6 (30 months later), demonstrate a strong homology between the two tumors.

### Analysis of recurrent alterations observed in patient tumors and xenografts

**Figure 5:**
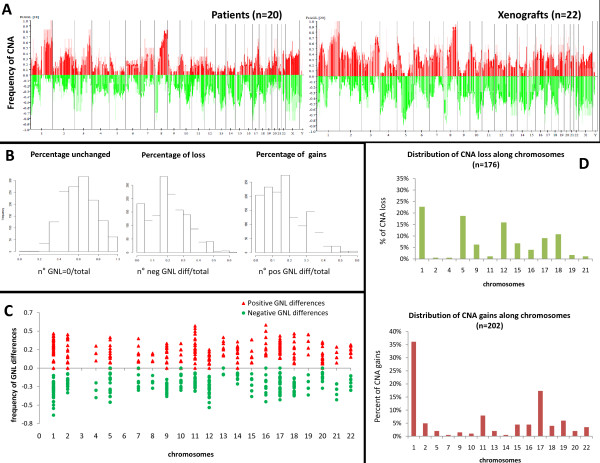
**Representation of differences in CNA between patient tumors and xenografts**. **A**, frequencies of genome copy number gains and losses plotted as a function of genome location in the patient tumors (left) and xenografts (right). Frequencies are based on GNL status and colors are calculated from the proportions of profiles without missing values. **B**, Frequency distributions plots of unchanged, positive and negative GNL differences. **C**, Frequency of CNA alterations distributed along chromosomes. Frequencies were calculated as the difference between patient tumor and xenograft GNLs. Negative frequencies represent DNA losses, while positive frequencies represent gains in xenografts. Only segments present in 10 or more pairs are shown (*n *= 992). **D**, Distribution of recurrent CNA gains (lower figure) and losses (upper figure) along chromosomes (frequency calculated for GNL differences occurring in at least 30% of pairs, *n *= 202 and 176, respectively). CAN, copy number alteration; FrAGL, Frequency of Amplicon, Gain and Loss; GNL, gain, normal, loss.

CNA frequency plots were analyzed for differences between patient tumors and xenografts (Figure 5A). The patient tumor plot showed highly rearranged profiles, reflecting the intrinsic genetic instability of BCs. This general CNA frequency pattern was reproduced in the xenograft plot with additional alterations observed for several chromosomes. To analyze the chromosomal regions that differed between xenografts and patient tumors, a "GNL difference" was calculated for each chromosomal segment: a segment was defined as a region with a constant copy number for all samples. The 1,351 segments analyzed are listed in Additional file 1, Table s1 and the frequency distributions of neutral, positive and negative GNL differences are shown in Figure 5B. A positive GNL difference indicates an increased copy number in the xenograft versus the patient tumor, while a negative GNL difference indicates a decreased copy number. Differences were calculated for 20 pairs corresponding to 18 patients and 20 xenografts (xenografts were generated from the primary tumor and, in addition, from a metastasis in two patients) and regions present in at least 10 comparisons were analyzed. The chromosomal distribution of these differences (corresponding to 992 segments) is illustrated in Figure 5C. The analysis was restricted to chromosomal segments showing negative or positive GNL differences in at least 30% of pairs (and present in at least 10 comparisons) in order to identify recurrent changes. It showed that 178 regions were lost and 202 regions were gained (Additional file 1, Table s2 and Table s3, respectively). The chromosomal distribution of these recurrent changes (Figure 5D) shows that gains mainly involved chr 1 and chr 17 (35% and 17%, respectively), while CNA losses occurred in chr 1, chr 5, chr 12 and chr 18 (23%, 19%, 16%, respectively). The chromosomal regions involved in recurrent gains and losses are shown in Table 2. Some regions (chromosomes 1p, 15q, 16p, 18p) were involved in both gains and losses, while other regions were mainly associated with either gains or losses.

**Table 2:** Chromosomal regions with DNA copy number differences between patient tumors and xenografts

Gain	Loss
1p22-36	1p12-36
2p16	1q21-q34
2p21-p24	2p11-q11
5p15	4p16
7q22	5q11-q31
9q34	9p23-24
10p15	9q13-32
11p13	11p15
11q12-q14	11q22
13q13	12p11-p13
13q33-q34	12q21
14q11	15q24-q26
15q22-q25	16p13
16p13	17p11-13
17p11-q11	17q23-q24
18p11	18p11
19q13	18q12-q22
20q11	19p11-p12
22q12-q13	19q22

### Molecular subtype classification

The molecular classification of patient tumors and xenografts was determined using 306 genes that allow for discrimination between the five BC molecular subtypes (Luminal A and B, Basal, HER2, Normal); these genes were defined and validated by Hu *et al*. [16]. Matching the UniGene ID (Build204) gene list to the HG-U133 Plus 2.0 Affymetrix^© ^platform annotation identified a total of 296 (729 probe sets) of the 306 genes. In a group of 28 tumors (including 10 patient tumors and 18 xenograft tumors at various later generations *in vivo*), 12 samples were classified as Basal, 6 as Luminal A, 3 as Luminal B and 2 as Normal (Table 3). Spearman correlation coefficients were less than 0.20 for seven of these tumors and the corresponding samples were consequently considered "unclassified". In 6 of the 10 comparisons, the molecular signature was concordant and in two cases the patient's tumor was classified as Normal and the xenograft was classified as Basal.

**Table 3:** Molecular subtype classification of primary tumors and corresponding xenografts at later generations

Tumor code	Origin	Molecular subtype
**HBC(x)-3**	patient	LumA
	xenograft p2	LumB
	xenograft p6	LumA
**HBC(x)-5**	patient	LumA
	xenograft p2	LumB
	xenograft p5	LumB
**HBC(x)-6**	patient	Basal
	xenograft p0	Basal
	xenograft p6	Unclassified
**HBC(x)-7**	patient	Unclassified
	xenograft p7	Unclassified
	xenograft p9	Unclassified
**HBC(x)-10**	xenograft p4	Basal
	xenograft p8	Basal
	patient	Basal
**HBC(x)-13**	patient	LumA
	xenograft p6	Unclassified
**HBC(x)-15**	patient	Normal
	xenograft p1	Basal
	xenograft p5	Basal
**HBC(x)-12A**	patient (primary tumor)	Normal
	xenograft (primary) p8	Unclassified
	xenograft (primary) p5	Basal
**HBC(x)-12B**	patient (metastasis)	Unclassified
	xenograft (meta) p3	Basal
	xenograft (meta) p6	Basal
**HBC(x)-19**	patient	LumA
	xenograft	LumA

HBC, human breast cancer; HBC-n, HBC primary tumor n; HBCx-n, HBC xenograft tumor; LumA, luminal A; LumB, luminal B; meta, metastasis; P, passage

### Gene expression analysis

**Figure 6:**
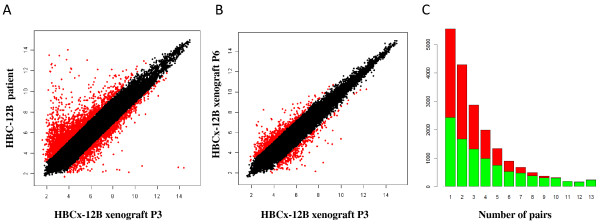
**Gene expression analysis by Affymetrix GeneChip probe arrays**. **A**, Variation of gene expression between the primary HBC-12B tumor and the corresponding xenograft. Scatter plot representing the gene expression of 29,683 probe sets (primary tumor versus xenograft p3). red dot, probe sets with high residual values. **B**, Variation of gene expression between xenograft at passage 3 and passage 6. **C**, Number of overexpressed (red) and underexpressed (green) genes between patient tumor/xenograft pairs HBC, human breast cancer; HBC-n, HBC primary tumor n; HBCx-n, HBC xenograft tumor n.

Linear regression analysis was performed to study the global gene expression pattern in xenografts *versus *primary tumors. An example of the correlation between gene expression patterns is shown in Figure 6. Probe sets that were not differentially expressed fitted a linear model (black dots), while red dots represent extreme residual values (overexpressed or under-expressed). The gene expression scatter plot between the patient tumor and the xenograft indicates that the great majority of probes were not differentially expressed (Figure 6A). Linear regression analysis performed of xenografts from different tumor passage (passage 3 versus passage 6) showed very few probe sets with differential expression (Figure 6B), indicating that gene expression did not undergo any major changes during the course of mouse-to-mouse grafts. Analysis of all paired samples (primary tumor vs corresponding xenograft) showed a significant decrease in the number of probe sets differentially expressed exclusively in one pair (5,530 out of 29,683; 18.6%) or common to 13 pairs (225 out of 29,684; 0.7%). A similar, significant trend was observed for the proportion of overexpressed versus under-expressed genes (Chi-square test, *P *= 1e-16) with a decreasing number of overexpressed genes when comparing the list of probe sets exclusive to one pair (3,114 out of 5,530; 56.3%) to the list of probe sets common to 13 pairs (6 out of 225; 2.6%) (Figure 6C). Gene Ontology analysis was performed on four probe set lists, that is, common to 1 to 3 pairs, 4 to 6 pairs, 7 to 9 pairs and 10 to 13 pairs (Additional File 2, Tables S1, S2, S3 and S4). A significant enrichment in annotations corresponding to the immune system, response to external stimuli, response to wounding, cell adhesion, inflammatory response, blood vessel formation, skeletal development and cell motility was observed in the group of probe sets common to 10 to 13 pairs (Table 4 and Additional file 3, Table S1). The group common to one to three pairs was mainly enriched in annotations corresponding to protein transport, messenger RNA processing, RNA splicing, cell adhesion, regulation of cell proliferation, regulation of cell death and apoptosis, but no annotation related to the immune system was identified. Analysis of these ontology annotations in the four groups showed an interesting pattern. Significant annotations in the first group progressively became non-significant in the second and third groups and, inversely, significant annotations in the fourth group progressively became non-significant.

**Table 4:** David Enriched Gene Ontology Categories in Genes Expressed Differently between patient tumors and xenografts

Gene Ontology Biologic Process	Count	%	List Total	Fold Enrichment	Benjamini
immune response	98	16.2	479	4.01	8.12E-30
cell adhesion	94	15.6	479	3.79	9.26E-27
biologic adhesion	94	15.6	479	3.79	6.90E-27
response to wounding	80	13.2	479	4.26	7.08E-26
defense response	72	11.9	479	3.31	1.51E-16
inflammatory response	49	8.1	479	4.26	7.35E-15
blood vessel development	41	6.8	479	4.73	1.62E-13
vasculature development	41	6.8	479	4.61	2.48E-13
extracellular matrix organization	26	4.3	479	7.06	4.28E-12
positive regulation of immune system process	35	5.8	479	4.15	8.12E-10
extracellular structure organization	28	4.6	479	4.85	4.15E-09
wound healing	30	5.0	479	4.44	6.13E-09
blood vessel morphogenesis	31	5.1	479	4.15	1.38E-08
positive regulation of response to stimuli	32	5.3	479	3.83	4.73E-08
regulation of response to external stimulus	26	4.3	479	4.62	5.46E-08
taxis	26	4.3	479	4.59	5.87E-08
chemotaxis	26	4.3	479	4.59	5.87E-08
angiogenesis	25	4.1	479	4.77	5.70E-08
regulation of cell activation	27	4.5	479	4.36	7.17E-08
regulation of cell migration	26	4.3	479	4.34	1.61E-07
skeletal system development	36	6.0	479	3.19	3.25E-07
regulation of locomotion	27	4.5	479	3.97	4.70E-07
regulation of cell motion	27	4.5	479	3.95	5.01E-07
regulation of T cell activation	21	3.5	479	5.07	4.87E-07

Enriched Gene Ontology Categories in Genes Expressed Differently between patient tumors and xenografts and common to 10 to 13 pairs (first 25 categories represented). BP, biologic process

For instance, gene expression analysis performed on xenografts after multiple passages identified only 11 probe sets (0.03%) differentially expressed in more than 50% of the paired samples (data not shown).

Altogether, these data show that xenografts display a relatively similar gene expression profile compared with their corresponding human tumors.

## Discussion

The aim of the present study was to analyze the molecular profiles of a panel of human BC xenografts directly transplanted from patient tumor samples. Tumors transplanted into a host animal are subject to different stroma and selection pressures, which can impact on the tumor's molecular profile. We, therefore, compared the genomic and gene expression profiles of BC xenografts with their corresponding patient tumors. As illustrated by histologic and immunohistochemical examinations, and as previously reported, these xenografts maintained the biologic markers of the patient's primary tumors as well as their microscopic morphology [8].

Characterization and comparison of CNA and clustering analysis showed that the original genetic profile was generally maintained in the xenograft. Accurate quantification of homology is a complex procedure, as breast carcinomas display intratumor heterogeneity [19,20] and xenografts are obtained by selecting only a small fragment of the tumor sample after surgery. The correlation between CNA profiles of pairs of xenografts and primary tumors was noteworthy, being greater than 0.5 for most of the paired samples (16/18). The similarity between genomic profiles appeared to be greater for triple-negative and luminal tumors than for HER2+ tumors, which could be explained by the fact that Her2 positive tumors are less markedly altered and are composed largely of stromal cells (see Figure 1), leading to underestimation of the correlation coefficient calculated on tumor DNA alterations. Analysis of the genomic profiles showed that the majority of tumors had a concordant distribution of chromosomal gains and losses, and maintained amplification regions. However, an enrichment of genomic rearrangements was observed in the xenografts, as previously demonstrated by our team, as well as by other authors [8,21-23].

Analysis of recurrent changes observed in xenografts showed enrichment of CNA in certain chromosomes. The majority of these regions are known to be associated with chromosomal imbalances in BC cells derived from either tumor samples or cell lines. In the present analysis, negative differences corresponded to deletions that were not detected in the patient tumor or DNA gains that were lost in the xenograft. Conversely, positive values represented gains observed in xenografts that were not detected in the patient tumor or deletions present in the patient tumor but lost in the xenograft. The greater number of DNA rearrangements observed in the xenografts compared to the patient tumors (Figure 5A) suggests that the CNA differences observed were due to gains or losses occurring in xenografts and not present in the patient tumors, rather than losses and gains in the patient tumors that returned to a normal status in the xenograft. In addition, the majority of regions presenting high frequencies of CNA changes were associated with chromosomal imbalances in BC. This result is concordant with those of a recent paper that analyzed the chromosomal aberrations in a panel of nine patient-derived models of sarcoma. Kresse *et al*. showed that many CNA changes found in xenografts are frequently observed in sarcoma patients, suggesting that xenografts may in some way represent the genomic rearrangement intrinsic to tumor progression [23]. In the case of breast cancer, Ding *et al*. studied the pattern of genetic differences between a patient's tumor and the corresponding xenograft. Although their conclusions are based on analysis of a single patient's tumor, this study elegantly demonstrated that many of the mutations detected in the xenograft were also observed in brain metastases derived from the same patient [24]. The fact that genomic alterations are conserved for several years, without any major changes, as demonstrated by sequential CGH analysis after multiple passages *in vivo*, suggests that genomic profiles remain relatively stable over time despite new selection pressures and loss of human stroma. This finding was also demonstrated by clustering analysis, in which the similarity between different xenograft passages was greater than the similarity between the xenograft and the original tumor, indicating that selection of breast tumor cells at the time of the first tumor engraftment is the major source of genetic variability. This observation can be explained by a selective tumor cell process during *in vivo *transplantation. In addition, loss of the human stromal compartment in xenografts results in enrichment of human tumor cells, and consequently enrichment of DNA alterations not detected in the patient's tumor.

The amplifications present in the patient's tumors were generally conserved in the xenografts, except for the HBC-8 tumor, in which the changes were limited to DNA gains. This suggests that these genomic regions do not undergo any major variations after grafting tumors into immunocompromised mice. More than 40 (42) amplicons were detected in the panel of BC xenografts (8p11.2-p12, 8q24, 11q13.3, 17q12-q21 and 20q13.3). Several of them have been frequently described in BC patients and carry potential oncogenes, the overexpression of which may be important for initiating, survival and/or development of breast tumors [25,26].

In terms of gene expression, less than 20% of probe sets showed significant variations on a single comparison and these genes were not enriched in stroma-related components. Investigation of genes with recurrent changes (common to 10 to 13 pairs) identified a small group of 205 genes associated with stromal gene ontology annotations, consistent with changes in the tumor environment from human to mouse. Another study compared gene expression between two BC xenografts and patient tumors [21]. Bergamaschi *et al*. reported a significant variation in the expression of human extracellular matrix-related genes that were down-regulated in xenografts compared with primary tumors. Interestingly, we also found that about one-half of the genes that were down-regulated in xenografts were correlated with breast carcinoma prognosis (data not shown). This set of genes is enriched in the immune system and is composed of immune response-related genes. Inversely, the set of genes not correlated with prognosis was highly enriched in wound response, cell adhesion, blood vessel development, extracellular matrix organization and cell migration related genes. These results indirectly emphasize the major role of the stromal compartment and the immune system in the prognosis of breast carcinoma [27].

The gene expression profile data may provide important information on pathway activation or cellular targets for novel anticancer agents, and it may also contribute to the identification of genes affecting response to treatment. Additional gene expression studies and pathway signaling analyses will be necessary to complete the molecular characterization of BC xenografts, especially in the context of preclinical development of molecular targeted agents.

## Conclusions

In conclusion, this panel of human BC xenografts, maintained by grafting fresh fragments through sequential passages, demonstrates that xenograft breast tumors reflect the general genetic profile of the patients' tumors. The genomic and gene expression profile differences observed were consistent with the high grade of genetic instability of BC, and loss of the human stromal component. In addition, studying tumors after various passages *in vivo *showed that these models conserve a high degree of genomic and gene expression stability over time.

These analyses support the use of the primary BC xenografts as preclinical models to study the effect of new anticancer drugs and to identify biologic factors associated with drug response.

## Abbreviations

aCGH: array comparative genomic hybridization; BAC: bacterial artificial chromosome; BC: breast cancer; CNA: copy number alterations; DAVID: Database for Annotation, Visualization and Integrated Discovery; ER: estrogen receptor; GNL: gain, normal, loss; HER2: human epidermal growth factor receptor 2; HBCx: human breast cancer xenograft; PAC: P1-derived artificial chromosome; PR: progesterone receptor.

## Competing interests

The authors declare that they have no competing interests.

## Authors' contributions

FR was responsible for microarray data analysis and interpretation, and CG was involved in CGH experiments and analysis, both contributed to writing the paper. CL and PG helped with statistical analysis of array CGH data. CD and DG performed RNA preparation and microarray experiments. NA contributed to array CGH experiments. FA and LdeP were involved in the *in vivo *experiments. MFP and PC were involved in the study design and clinical supervision of the project. AVS, PdeC and JJF were involved in sample collection and pathological examination of the tumors. OD took part in array CGH data analysis and contributed to the study design. SRR and DD contributed to management of the xenograft project. EM was the principal investigator and instigated the study, supervised the experimental and scientific design, and wrote the paper. All authors have read and approved the manuscript for publication.

## Supplementary Material

Additional file 1 **Supplementary Tables 1, 2 and 3**. Table S1: Status assignment of chromosome segments. GNL (loss, normal, gain of chromosome copy number). Start/end positions, size (Mb) and chromosome assignment are associated with each segment. Table S2: Chromosomal segments showing negative GNL differences in at least 30% of pairs (and present in at least 10 comparisons). Table S3: Chromosomal segments showing positive GNL differences in at least 30% of pairs (and present in at least 10 comparisons). Click here for file

Additional file 2 **Supplementary Tables 1, 2, 3 and 4**. Table S1: Gene Ontology (GO) analysis performed on four Probe Sets; that is, common to 1 to 3 pairs. Table S2: Gene Ontology (GO) analysis performed on four Probe Sets; that is, common to 4 to 6 pairs. Table S3: Gene Ontology (GO) analysis performed on four Probe Sets; that is, common to 7 to 9 pairs. Table S4: Gene Ontology (GO) analysis performed on four Probe Sets; that is, common to 10 to 13 pairs. Click here for file

Additional file 3 **Supplementary Table 1**. List of genes differentially expressed between patient tumors and xenografts (common to 10 to 13 pairs). Click here for file
